# Progress of Veterinary Science

**Published:** 1881-04

**Authors:** 


### PROGRESS OF VETERINARY SCIENCE.

---

Nitrite of Amyl has been found to work well as an antidote to carbolic acid poisoning. In the case of a horse accidentally poisoned by carbolic acid, the nitrite was used in 30 to 60 drop doses, and great relief was obtained.

Composition and Amount of Urine of the Horse.—Volume in 24 hours, 6 litres (2 quarts); color, yellow amber; reaction, alkaline; density, 1.023; urea, 38 grams (6.3 per litre); hippuric acid, 132 grams (22 per litre); mineral salts, 35 to 40 grams per litre.

Chloral as a Sedative for Horses.—There is reason to suppose that chloral is sometimes given by coachmen to their horses to render them less spirited and more easy to drive. In a case which recently occurred at Bordeaux, the plan answered its end admirably; for a very fiery horse which formerly could hardly be kept in hand, became, after a few days, quiet and gentle. This practice, which has grown to be a serious abuse in some quarters, should be looked to by veterinary surgeons.

Treatment for Piles in Horses.—Drench the animal with one ounce of spirits of turpentine in one pint of castor oil and one of warm water, hot enough to keep the oil thin, twice weekly. Apply the following to the piles with a small glass syringe, or, if not too deep in, with the finger. Tannin, four grains; sulphate of zinc, sugar of lead, chloride of zinc, of each four grains; laudanum, two ounces; olive oil, two ounces. Apply daily once or twice.—*Indiana Farmer.*

How to Examine the Horse's Eye.—Examine the eye first, when the horse stands with his head to the manger. Look carefully at the pupil of the eye in the horse; it is of an oblong form; carry the size of the pupil in your mind, then turn the horse about, bring him to the bright light, and if the pupil of the eye contracts and appears much smaller than it was in the darker light, then you may be sure the horse has a strong, good eye; but if the pupil remains nearly the same size as it appeared in the darker light, the horse has a weak eye, therefore have nothing to do with him.—*National Live Stock Journal.*

TREATMENT OF "MOON BLINDNESS."—In connection with Prof. Moore's article on Moon Blindness, in another part of the JOURNAL, we give the following mode of treatment recommended by Dr. Paaren in the *Live Stock Journal:* The horse should be placed in a darkened stall, and after a meal or two of bran mash, given a physic, composed of five drachms of aloes, and one drachm of podophyllin, two drachms of nitre, and one drachm of capsicum. Bathe the eye thrice daily with warm water, by means of a soft sponge, and apply between the lids, with the aid of a small camel's-hair pencil, a portion of a mixture of half an ounce of Goulard's extract, one ounce of fluid extract of belladonna, and twenty-four ounces of distilled water. Externally, apply to the hollow space over the orbit of the eyes, once daily, a small portion of weak mercurial ointment. Give loosening food in small quantities. This disease is not permanently curable; it is apt to return again and again, and finally end in cataract, or a complication of pathological changes of a permanent nature. Each attack leaves the eye weaker, and partial or total blindness results sooner or later.

A NEW POISON IN PORK has been recently announced in England. It is thought to be produced by the presence of certain rod-shaped bacteria. The symptoms are severe diarrhœa and prostration.

TREATMENT OF TRICHINOSIS BY GLYCERINE.—Several cases of trichinosis have been reported in which recovery took place under the use of large doses of glycerine. The treatment of trichinosis by glycerine is based on the fact that glycerine, when applied to the living parasites, shrivels them up and kills them. The treatment has been employed largely in Germany with varying success. It is hardly probable that glycerine can do anything more than kill the trichinæ that are in the alimentary canal, and we doubt if it even does this.

TREATMENT OF HOG CHOLERA.—Hog cholera is said to be prevented in France by disinfecting the piggeries, and by giving in each full-grown pig's food a teaspoonful of *levenaijre phenique*, which is made of 2½ ounces of pure carbolic acid, and one gallon of common vinegar; and also by occasionally giving the pigs a dose of nitre or sulphate of soda.

A writer in the *Live Stock Journal* sends the following: Twelve grains of quinine to each hog weighing 250 pounds, or at the rate of five grains for each 100 pounds will, I think, prevent any hog or pig dying that is well enough to eat.

As will be seen elsewhere, Dr. Lindsay has been successful with chlorate of potash.

A NEW DISEASE DEVELOPED FROM HYDROPHOBIC SALIVA.—Pasteur, Chamberland, and Roux unite in arriving at the conclusion that a new disease can be developed from the saliva of a victim to hydrophobia. The germ which produces the disease is of the shape of a small rod constricted at the middle and imbedded in mucous matter. Strange to say, although closely resembling the microbe which causes chicken cholera, it has no effect upon fowls. The saliva experimented with was taken from a child that had died of a bite from a mad dog. Rabbits inoculated with a dilute solution of it, after suffering from loss of appetite, paralysis, swellings in the groin and in the axillæ, died within 36 hours, and other rabbits inoculated with the blood of those which had been poisoned with the saliva soon died also. Dogs similarly treated did not live more than two or three days, but they did not manifest any of the symptoms of rabies. Guinea pigs, although so like rabbits, seemed to escape almost all evil consequences. The experimenters are of the opinion that the disease is quite different from hydrophobia; but they have not as yet pushed their researches far enough to be able to say what precise connection exists between the two maladies.

Cure for Dog Distemper.—Two Views.—I see it stated in your valuable paper of January 13th, that there is no cure for dog distemper. In this Dr. Horne is mistaken. The remedy is common salt. Open the dog's mouth and put a lump of salt about the size of a hen's egg on his tongue, then close the dog's jaws together, and hold until the salt is dissolved and swallowed. If this does not give relief in a few hours, repeat the dose until the dog vomits freely.—*Country Gentleman.*

A cure for this is frequently asked for. There is no cure for distemper. Like scarlet fever, measles, small-pox, &c., it must run its course, and any man who assumes to cure any of those diseases (and many others similar) should receive the contempt due to arrogant ignorance. All these diseases are subject to skill and proper medication. All that the most scientific can do is to administer such medicines as will, with proper and judicious nursing, bridge over, so to speak, the crisis, and so bring it to a favorable termination. Nothing more can be done.

We endorse the last view, which is that of Dr. William Horne. Another correspondent gives the following directions for preventing the disease:

Feed puppies on milk and vegetables well cooked and salted; flour and meal (especially oatmeal) made into pudding or bread, till they are about nine months old, and they will rarely be affected with the distemper. Give a bone now and then to gnaw; also to mix soup with the bread, pudding or vegetables, is a good thing. My friends and self have thus raised many puppies for years past, and not a single case of dog distemper.—*Country Gentleman.*

Foot-rot is a very troublesome disease to the shepherds in wet or muddy places, or wet pastures. The cause is as follows: Between the hoofs of the sheep a small aperture may be seen, called the biflex canal, whose office it is to secrete an oily fluid for the purpose of lubricating the hide between the hoofs, it being called into action by every step the sheep takes in providing its food. When perpetually wet, or constantly dirty, the parts swell, and this secretion already spoken of is stopped, or retarded; therefore not only is the hide deprived of the oily secretion, but the secretion itself becomes an irritant of the glands which secreted it; hence inflammation is the consequent result; hence foot-rot, which, unless retarded and remedied very soon, destroys not only the hoofs, but the glands, and perhaps the coronary border which secretes the hoofs.—J. N. Navin, *Indiana Farmer.*

Dyspepsia in Hens.—Chickens are sometimes troubled with indigestion. The symptoms are dullness and fullness of crop. When in this condition they are said to be "crop-bound." If the contents of the crop seem to be of a liquid nature, pulverized charcoal is an excellent remedy; but if the crop is hard and solid, a surgical operation is the only means of relief, and unless the bird is a very valuable one this will not pay. A constant supply of charcoal and occasional feeds of raw unions will prevent indigestion.—*Indiana Farmer.*

Transmission of Tuberculosis.—According to M. Toussaint and M. Larrey, bovine tuberculosis is transmissible in the following ways: 1st, by the ingestion of tuberculous matters; 2d, through the teat of a tuberculous cow; 3d, through the blood by inoculation; and 4th, by simple contact.

According to M. F. Pench (*Comptes Rendus*) it is an undoubted fact that consumption can be transmitted to the human subject by the use of the milk of a cow affected by this disease. While we do not consider the latter point absolutely established, there is enough evidence of its truth to make it extremely important.

Diseases of Sheep.—Some very interesting facts regarding this subject were given by Dr. J. N. Navin, in an address before the Indiana Wool and Drovers' Association, printed in the *Indiana Farmer*, though we are hardly prepared to accept Dr. N.'s statement that malaria has so powerful an influence in causing disease among sheep. The sheep, he says, is naturally the healthiest, though the tenderest of all domestic animals, scarcely ever getting sick except from some great provocation, malarial influence having more to do with it than all other causes combined. In Europe the sheep is subject to about twenty-five diseases, none of which are incident without cause or provocation, except, perhaps, thrush in the mouth, or blain. With these two exceptions, all others are the result of bad treatment, or of malarial influence. Foot disease, the rot, and dropsy are the result of malaria and pasturing upon wet and marshy lands. Diarrhœa and dysentery are the effect of feeding, or of some affection of the liver by malaria. Water on the brain, epilepsy, apoplexy, hydatid on the brain, and lockjaw, have causes the same as in other domestic brutes. Bots in the sinuses of the head are the larvæ of the fly, so-called, but smaller than those which horsemen dread so much. Bronchitis, lung fever, colic and inflammation of the brain are also manufactured diseases, or the effect of treatment, or usage.

Is Charbon Transmitted by Plants and the Soil?—Dr. Colin has been examining experimentally the claims of Dr. Pasteur that charbon can be transmitted by plants which may be grown in the place where charbonized bodies have been buried. He had taken the opportunity of burying sixty animals, dying from charbon, in the soil at varying depths. The grass and shrubs growing on the soil have been eaten by rabbits without producing charbon. The earth taken from immediate contact with the carbonized body has been mixed with forage and used as food for animals, without any result, and the water in which this earth has been dissolved has been mixed with food and given to animals. It has also been inoculated, but in not a single case did charbon result, whence he concludes charbon is not transmissible by plants or the soil.—*Chicago Medical Review.*

Roup in Fowls.—A correspondent of the *Indiana Farmer* writes: "Our chickens are affected with a disease unlike anything I have ever seen or heard of before. It affects their eyes, feet and mouth. In some of the fowls it makes its appearance first in their eyes, in others it will appear first in their mouths. Their eyes will become closed, the lids stick together. On opening them we find a milky matter, also pieces of thick clotty matter will adhere to the ball and lids, which may be scraped off, and leaves the places raw and bloody. The roof of the mouth, the tongue, and the mouth of the windpipe have this same kind of thick matter on them. They seem to be eating sores. Their windpipe sometimes will become so swollen as to make it almost impossible for them to breathe. It is a lingering disease; sometimes they live for two or three weeks. Occasionally one will get over it."

In answer to this, the Editor says: The disease is no doubt an aggravated form of roup. Separate the diseased fowls from the well, putting them in a dry, warm place, and give a dose of castor-oil every morning for a week. Mix pepper with their feed, which should be soft and warm. Wash head and eyes and inside of mouth and nostrils with vinegar in water. It is cleansing and healthy.

Black-leg (External Charbon), not Anthrax.—Another Protective Virus.—Dr. D. E. Salmon (*Cultivator and Country Gentleman*), reviewing the history of the above two diseases, is of opinion that they have been proved distinct affections.

The disease known as black-leg is generally fatal. It makes its appearance suddenly, with dullness, loss of appetite, and the formation of an irregular swelling, not plainly circumscribed, and appearing on the trunk, neck, under the jaw, or on one of the legs. This swelling increases with astonishing rapidity, invading the muscles and the inter-muscular interstices. At first it is homogeneous and very painful, but becomes by degrees insensible, crepitating and resonant in its centre; the tissues forming it are black and friable, and on incision, there escapes blood, which is at first red, then black, and finally the escaping liquid is a frothy serum, with a large quantity of gas. While the local symptoms are developing, the general condition is aggravated, the temperature rises at first, then falls, and the animal dies in thirty-six to forty-eight hours.

In the areola tissue and serum of the tumor, and, at times, in the lymphatic glands, kidneys, lungs, and even blood, are found bacteria, existing as bright oval corpuscles, single or united end to end, to the number of two or three, and also short rods moving in all directions. These rods are shorter and broader than those seen in anthrax; they are very motile, rounded at their two extremities, and are nearly always provided near one of these, never in the middle, with a clear nucleus. These bacteria are different from those of anthrax.

Further, by properly preparing the virus of the disease, and injecting it into the veins, there results simply dullness, loss of appetite and fever, the temperature not rising more than 1.9° C. (3.4° F.) in extreme cases; and these symptoms remain but one, two, or three days, disappearing generally more rapidly with the calf and goat than with the sheep. Such inoculation does not produce local swellings when proper precautions are taken to prevent the virus touching the areolar tissue at the point of inoculation.

Finally, such intra-vascular inoculation enable the subjects to resist subsequent injections of virus into the areola tissue or muscles; instead of the inflammatory tumor ending in death, which follows such inoculation in susceptible animals, there is only an abscess, in which, however, the bacterium preserves its activity.

Thus we have another method of preventive inoculation. Within the last year such methods have been discovered for three distinct contagious diseases, viz., anthrax fever, symptomatic charbon, and chicken cholera.

It should be remembered, in connection with these experiments and alleged discoveries of the French investigators, that they still lack corroboration, and the role of the bacteria in the different diseases is by no means demonstrated to be a fundamental and essential one.

